# Lysine-specific demethylase (LSD1/KDM1A) and MYCN cooperatively repress tumor suppressor genes in neuroblastoma

**DOI:** 10.18632/oncotarget.3990

**Published:** 2015-05-04

**Authors:** Stefano Amente, Giorgio Milazzo, Maria Cristina Sorrentino, Susanna Ambrosio, Giacomo Di Palo, Luigi Lania, Giovanni Perini, Barbara Majello

**Affiliations:** ^1^ Department of Biology, University of Naples, Naples, Italy; ^2^ Department of Molecular Medicine and Medical Biotechnologies, University of Naples, Naples, Italy; ^3^ Department of Pharmacy and Biotechnology, University of Bologna, Italy; ^4^ CIRI Health Sciences and Technologies (HST), Bologna, Italy

**Keywords:** MYCN, Neuroblastoma, LSD1, Transcription

## Abstract

The chromatin-modifying enzyme lysine-specific demethylase 1, KDM1A/LSD1 is involved in maintaining the undifferentiated, malignant phenotype of neuroblastoma cells and its overexpression correlated with aggressive disease, poor differentiation and infaust outcome. Here, we show that LSD1 physically binds MYCN both *in vitro* and *in vivo* and that such an interaction requires the MYCN BoxIII. We found that LSD1 co-localizes with MYCN on promoter regions of CDKN1A/p21 and Clusterin (CLU) suppressor genes and cooperates with MYCN to repress the expression of these genes. KDM1A needs to engage with MYCN in order to associate with the CDKN1A and CLU promoters. The expression of CLU and CDKN1A can be restored in MYCN-amplified cells by pharmacological inhibition of LSD1 activity or knockdown of its expression. Combined pharmacological inhibition of MYCN and LSD1 through the use of small molecule inhibitors synergistically reduces MYCN-amplified Neuroblastoma cell viability *in vitro*. These findings demonstrate that LSD1 is a critical co-factor of the MYCN repressive function, and suggest that combination of LSD1 and MYCN inhibitors may have strong therapeutic relevance to counteract MYCN-driven oncogenesis.

## INTRODUCTION

Neuroblastoma (NB) is a pediatric tumor with poor outcome and highly refractory to therapeutic treatment. The molecular bases of NB development and progression are still poorly understood. The best-characterized genetic markers include amplification of the proto-oncogene MYCN, amplification and mutation of ALK gene and chromosomal alterations [1-7]. Classical risk factors include the age at diagnosis, MYCN amplification and stage of the disease. MYCN is a member of the MYC family (MYC, MYCN and MYCL) proteins that are basic Helix-Loop-Helix Leucine Zipper (bHLHZip) transcription factors, which forms transcriptionally active hetrodimers with another bHLHZip protein called MAX [8-9]. Dimerization with Max endows Myc with sequence specific DNA binding ability, preferentially to sites containing the E-box sequence CACGTG. Activation of MYC oncogenes simultaneously coincides with global modifications in chromatin structure and subsequent robust changes in MYC targets gene expression. MYC proteins have been found to orchestrate epigenetic alterations by recruitment of higher order chromatin complexes that activate or repress transcription.

MYC/MYCN have been found to associate with different chromatin modifying complexes and their role in transcription depends on both histones tails modifications already present at promoters of its target genes and on the biochemical composition of protein complexes that MYC can recruit in different cellular environment [9, 10, 11, 12]. Large body of evidences suggest that supra-physiological expression of MYCN drives tumorigenesis by increasing the expression of cell cycle-related genes with a concomitant transcriptional silencing of genes involved in negative regulation of cell proliferation and transformation [13]. While the mechanisms by which MYCN can act as a transcriptional activator have been extensively studied, how MYCN can exert its transcription repression function is largely unknown [14].

It has been shown that MYCN can suppress gene expression by interacting with sequence-specific transcription factors such as SP1 and MIZ1 and by recruiting transcriptional co-repressors at the promoter sequences of suppressor genes such as TRKA, p75NTR, CDNK1A/p21[15]. Moreover, induction of mi-RNA encoding genes represents an alternative mechanism through which Myc represses gene expression [16]. Collectively, these studies indicate that the aberrant expression of MYCN can modify gene expression both via direct and indirect mechanisms.

It has been documented that MYCN inhibits gene expression of the putative tumor suppressor gene CLU (Clusterin) by direct binding to the non-canonical E box sequence within the regulatory region. There, it induces bivalent epigenetic marks and recruitment of repressive complexes such as Polycomb repressive complex 2 (PCR2) through physical interaction with the EZH2 subunit [17, 18].

Undifferentiated NB with MYCN aberrant expression have been found associated with elevated levels of EZH2 as well as with Lysine-specific demethylase 1 (LSD1) [18, 19]. LSD1 (also known as KDM1A and AOF2) is an amine oxidase that catalyzes lysine demethylation in a flavin adenine dinucleotide (FAD)-dependent oxidative reaction [20, 21]. LSD1 removes mono- and dimethyl groups from lysine 4 (H3K4) [20] and lysine 9 (H3K9) [22] of histone H3, and can also act on nonhistone proteins including p53, E2F1, and DNMT1 [23, 24, 25]. LSD1 was originally found to be part of the chromatin-modifying complex Co-Rest [26]. The REST/Co-Rest complex, which includes LSD1, as well as HDAC1/2, is recruited at promoters to repress transcription of neuronal-specific genes [19, 27]. The current scientific literatures points to a critical role for LSD1 in cancer cell biology [29] particularly in the maintenance of silencing of differentiation genes [29, 30, 31]. LSD1, in fact, occupies promoters of a portion of proneural genes that contain bivalent domains and chromatin regions containing both H3K4me2/H3K4me3 and H3K27me3 marks, where LSD1 controls the levels of H3K4 methylation in order to keep these genes silent [32]. LSD1 is strongly expressed in neuroblastomas, and overexpression has been shown to correlate with poor prognosis [19]. Moreover, several recent reports highlight the crucial role for LSD1 in inhibition of differentiation genes; recently the ubiquitin-proteasome E3 ubiquitin ligase, Jade-2 was identified as a major LSD1 negative regulator during neurogenesis that specifically targets LSD1 for degradation and promotes Neuroblastoma cell differentiation [33].

In this present study we explored the functional interaction between LSD1 and MYCN and how such an interaction may be critical for Neuroblastoma biology. We found that LSD1 can directly interact with MYCN in NB cells and cooperate with MYCN to repress the expression of genes involved in negative regulation of cell proliferation and transformation such as CDKN1A/p21 and the putative tumor suppressor gene CLU (Clusterin). Our findings suggest that the MYCN/LSD1 complex has a direct role in maintaining the epigenetic silencing of dedicated MYCN target genes. Pharmacological inhibition of either MYCN or LSD1 or combination of both drugs have significant effects in neuroblastoma cell cycle and viability partly through activation of apoptosis.

## RESULTS

### LSD1 interacts with MYCN

**Figure 1:**
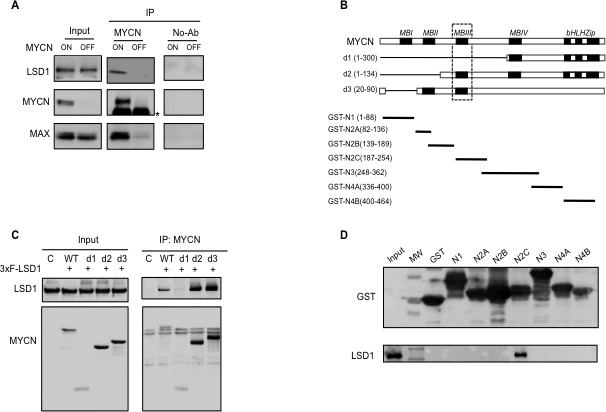
MYCN physically interacts with LSD1 **A.**, co-immunoprecipitation interaction between endogenous LSD1 and MYCN in Tet-21/N cells. Cell lysates from Tet-21/N cells Tetracycline-treated (6days) (MYCN-OFF) and untreated (MYCN-ON) were immune-precipitated with a MYCN antibody and a No-Ab sample was used as negative control. Western blot analysis was performed on immuno-purified extracts with MYCN, LSD1 and MAX antibodies as indicated; * indicates IgG. **B.**, schematic representation of MYCN deletion mutants d1, d2 and d3 used in the CoIP assay described in panel C and of GST-MYCN constructs used in GST-pull down described in panel D. The MYCN segments cloned in the GST expression vector are in black, and numbers indicate amino acid positions. **C.** MYCN-LSD1 interaction. 293T were cells co-transfected with an LSD1 expression vector together with different MYCN deletion expression vectors indicated in panel B. Extract from transfected cells were Immuno-precipitated with a MYCN antibody and analyzed by western blotting. **D.** Immobilized GST-MYCN polypeptides were incubated with equal amounts of extract prepared from HEK 293T cells transfected with the recombinant vector 3xFLAG-LSD1protein, separated by SDS-PAGE, and probed with an anti-LSD1 antibody.

It has been shown that LSD1 can reside in many different regulatory complexes involved either in repression or activation of gene transcription. We have previously shown that c-MYC interacts with LSD1 [34]. and since c-MYC and MYCN proteins share extensive structural and functional similarities we sought to determine whether LSD1 and MYCN can be associated in Neuroblastoma cells where MYCN is critical to the oncogenic process. To this end, co-immunoprecipitation analysis was carried out using the human Tet-21/N neuroblastoma cell line conditionally expressing MYCN under the control of a Tet-Off (tetracycline) promoter [35]. Protein extracts were prepared from tetracycline treated (MYCN-OFF) and untreated cells (MYCN-ON) and subjected to immunoprecipitation with an anti-MYCN monoclonal antibody. Next, immunoprecipitated complexes were analyzed by Western blotting for the presence of MYCN, LSD1 and MAX, respectively. Results in Figure 1A show that the immunoprecipitated complex from Tet-21/N with high MYCN, (MYCN ON), contained both the LSD1 and MAX proteins, thereby indicating that high levels of MYCN can form a complex with LSD1, and that binding with MAX is not mutually exclusive. Consistently, the association between MYCN and LSD1 was barely detectable in low MYCN expressing cells. We next sought to identify the MYCN regions responsible for the interaction with LSD1. To address this aim we adopted two independent approaches. First HEK 293T cells were co-transfected with expression vectors encoding the human full-length LSD1 together with a series of independent constructs expressing different MYCN deletion mutants: d1(aa1-300), d2(aa1-134) and d3(aa 20-90) described in Figure 1B. Mutant proteins were immunoprecipitated with an anti-MYCN specific antibody and analyzed by Western blotting to detect LSD1. As shown in Figure 1C, LSD1 was not immunoprecipitated by the d1 mutant lacking the 1-300 aa region while it was immunoprecipitated when the d2(1-134), and d3(20-90) mutants were employed. The comparison of the three mutants suggests that the MYCN region required to interact with LSD1 may reside between aa 134 and aa 300 which includes the MYCN BoxIII (Figure 1B and 1C). To corroborate this finding we carried out an *in vitro* GST pull-down assay using GST-MYCN constructs expressing different domains of MYCN indicated in Figure 1B. Purified GST-MYCN fragments were immobilized onto agarose beads and subsequently incubated with nuclear protein extracts obtained from HEK 293T cells transfected with a 3xFLAG-LSD1 construct. Results show that LSD1 can specifically interact with the region of MYCN between aa 187-254, encompassing the MYCN BoxIII (Figure 1D). Taken together, these data demonstrate that MYCN and LSD1 can associate both *in vivo* and *in vitro* and that the BoxIII domain of MYCN is likely required for direct interaction with LSD1.

### LSD1 inhibition releases MYCN-mediated repression of CDKN1A/p21

Previous findings demonstrated that LSD1 inhibition blocks Neuroblastoma cell proliferation [20]. Because MYCN binds and regulates pivotal cell cycle controlling genes such as CDKN1A/p21 and p53 [14, 15, 36], we investigated the relative levels of these proteins in relation to MYCN and LSD1 expression in the conditional MYCN expressing Tet-21/N cells in the presence or absence of functional LSD1. The relative expression levels of CDKN1A/p21 and p53 were determined in both MYCN-OFF and MYCN-ON cells as a function of active or inactive LSD1. Inhibition of LSD1 activity was obtained using either the tranylcypromine (TCP) inhibitor or by protein depletion using sequence-specific siRNA (siLSD1).

**Figure 2:**
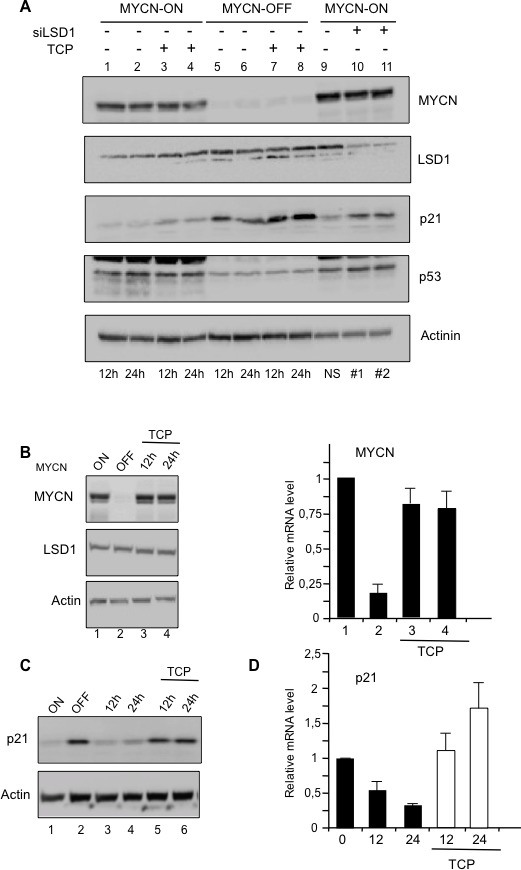
**A.** Relative expression levels of MYCN, LSD1, p21, and p53 proteins were determined by Western blot analysis with the indicated antibodies at 12 and 24 hrs after TCP treatment (lanes 3, 4, 7, 8) in MYCN-ON (lanes 1-4) and MYCN-OFF (lanes 5-8) Tet-21/N cells. MYCN-ON cells were treated with control siRNA (lane 9) or with two concentrations (20nM lane 10 and 100nM lane 11) of specific LSD1 silencing by siRNA. Actinin was used for loading normalization. **B.** MYCN-ON cells, lane 1, were treated for 6 days with tetracycline and these cells are referred as MYCN-OFF, lane 2. MYCN-OFF cells were depleted of tetracycline and treated with TCP. Cells cultivated for 12 and 24hrs lane 3 and 4, are collected for protein and mRNA analysis. **C.** TCP relieves p21 protein expression. MYCN-OFF cells were depleted of tetracycline for 12 and 24 hrs in absence, lane 3,4 and presence of TCP, lane 5, 6. **D.**, p21 mRNA expression. As in **C.**, MYCN-OFF cells (0) were depleted of tetracycline for 12 and 24 hrs in absence and presence of TCP.

MYCN, LSD1, p21, and p53 protein levels were determined by Western blotting analysis at 12 and 24 hrs after TCP treatment in both high and low MYCN conditions (Figure 2A). Consistent with previous findings, higher levels of p53 protein were observed in MYCN-ON cells compared to MYCN-OFF cells. There, p53 expression was unaffected by LSD1 inhibition. Furthermore CDKN1A/p21 expression levels inversely correlate with that of MYCN (lane 1 to 4 compared to lane 5 to 8). More importantly the TCP treatment (lanes 3-4 and 7-8) and the LSD1 depletion (lanes 9-11) caused de-repression of CDKN1A/p21 also in presence of MYCN over-expression, thus unveiling a decisive role of LSD1 in MYCN-driven repression of CDKN1A/p21.

To corroborate these findings, MYCN-ON cells in Figure 2B were treated for 6 days with tetracycline to lowering MYCN levels (MYCN-OFF), next they were grown in absence of tetracycline to reactive MYCN but also kept for 12 and 24 hrs in the presence of TCP to inhibit LSD1 function. Figure 2B, shows that both MYCN protein and mRNA levels were strongly induced at 12 hrs after tetracycline removal. Conversely, LSD1 expression was largely unaffected. Activation of MYCN coincided with CDKN1A/p21 repression, which was instead relieved by the TCP treatment (Figure 2C). Overall, these results reveal an unexpected role of LSD1 in MYCN-mediated repression of CDKN1A/p21 and demonstrate that LSD1 inhibition de-represses p21 expression in presence of high levels of MYCN, independently from p53 expression.

### MYCN and LSD1 co-localize at CDKN1A promoter

**Figure 3:**
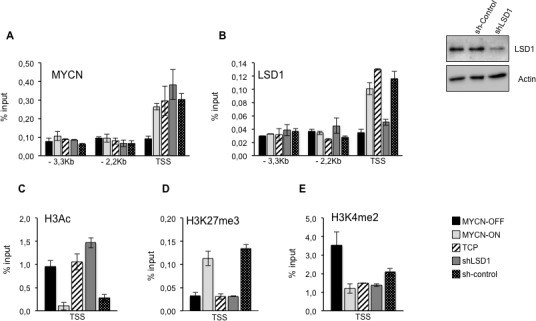
**A, B.** LSD1 and MYCN bind and repress p21. Chromatin immunoprecipitation assays. MYCN, LSD1, antibodies were used in IPs. Immunoprecipitated samples were analyzed by qPCR using specific primers for CDKN1A promoter Transcriptional Start Site (TSS) and two upstream regions (−3.3 and −2,2 KB. MYCN-OFF (black bars), MYCN-ON (light gray bars), MYCN-ON TCP treated (slanting bars), MYCN-ON shLSD1 (dark gray bars) MYCN-ON sh-control (dotted bars). LSD1 silencing in Tet-21/N cells transduced with shLSD1 and with sh-control was assayed by western blot shown in upper right. **C.**, **D.** and **E.**, Histone modifications at p21 promoter. H3Ac, H3K27me3 and H3K4me2 antibodies were used in IPs. Immunoprecipitated samples were analyzed by qPCR using specific primers for CDKN1A promoter Transcriptional Start Site (TSS). Data from three independent Chromatin-IP assays were used to make % of input graphs presented along with standard deviations, *n* = 3.

The findings reported above strongly suggest that LSD1 cooperates with MYCN to repress CDKN1a/p21 expression. We assessed the relative binding of MYCN and LSD1 to the CDKN1A/p21 gene in MYCN-OFF and MYCN-ON cells by chromatin immune-precipitation. Moreover, the relative MYC and LSD1 binding was analyzed in MYC-ON cells treated with TCP or silenced for LSD1 expression. The immunoprecipitated chromatin samples were subjected to qPCR using primers corresponding to the indicated regions of the CDKN1A/p21 gene. As shown in Figure 3A and 3B, MYCN and LSD1 were both detected at the transcriptional start site (TSS) of the CDKN1A gene but not in the distal region (−3,3kb and −2,2kb), indicating that CDKN1A/p21 promoter was directly bound by the MYCN/LSD1 complex. As expected, MYCN binding was enhanced in MYCN-ON cells. Nevertheless its binding to CDKN1A/p21 was unaffected by TCP or LSD1 depletion implying that MYCN binding does not require LSD1. In contrast, LSD1 binding was drastically reduced in MYCN-OFF cells, suggesting that LSD1 needs to engage with MYCN in order to associate with the CDKN1A/p21 promoter.

To better understand how MYCN/LSD1 complex can affect chromatin organization at CDKN1A/p21 promoter, we analyzed three different histone modifications, H3 pan-acetyl (H3Ac), H3K27Me3 and H3K4Me2 around the CDKN1A/p21 TSS promoter region. Figure 3C shows a strong reduction of H3 acetylation, in samples with high levels of MYCN whereas both LSD1 silencing (shLSD1) and inhibition by TCP determine a significant increase in H3 acetylation. As a marker of transcriptional repression we analyzed Lysine 27 tri-methylation of Histone H3. Data presented in Figure 3D show an almost 3 fold increase of H3K27Me3 histone marker levels on the p21 promoter region as a function of the increased presence of MYCN, whereas both LSD1 silencing or its inhibition (TCP) determine a decrease of the marker. Finally, chromatin-IP assays were also performed on di-methylated Lysine 4 of histone H3 (Figure 3E). As expected, high MYCN levels determine an almost 3 fold decrease of the H3K4Me2 on p21 TSS region. Consistently with data reported previously [37] both inhibition and repression of LSD1 do not significantly affect H3K4Me2 signature at TSS level of CDKN1A/p21. Our findings suggest that a MYCN/LSD1 complex binds to and represses the CDKN1A/p21 promoter, and that reduction of MYCN levels as well as LSD1-knockdown determine re-activation of CDKN1A/p21 expression.

### LSD1 and MYCN cooperate to repress Clusterin expression

**Figure 4:**
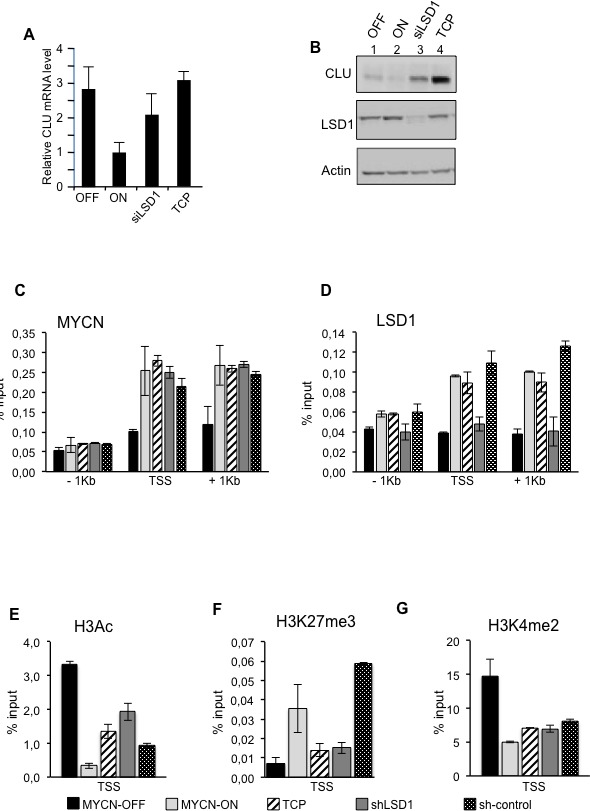
LSD1 and MYCN cooperatively repress CLU expression CLU gene expression was analyzed by qRT-PCR, **A.** or by western blot, **B.** using samples prepared from MYCN-OFF cells and MYCN-ON cells untreated and treated with TCP or siLSD1 as indicated. **C. D.** MYCN and LSD1 binding to CLU chromatin. Cell treatments are indicated at the bottom of the figure and described in the legend of Figure 3. qPCR was performed with primers for CLU TSS, −1kb and +1kb. **E. F.** and **G.**. Histone modifications at CLU gene; ChIPs were carried out using the indicated antibodies and analyzed with primers encompassing the TSS region. Data from three independent CoIP assays and presented along with standard deviations, *n* = 3.

It has been recently shown that MYCN interacts with EZH2, a component of the Polycomb repressor complex PRC2 and that the MYCN/EZH2 complex represses the tumor suppressor gene Clusterin CLU [17]. Because LSD1 can form complexes with both MYCN and EZH2, we hypothesize that LSD1 could contribute to CLU gene expression. To investigate the function of LSD1 in CLU regulation we inhibited LSD1 in MYC-ON cells with TCP or siLSD1 and compared CLU mRNA and protein expression to that observed in MYCN-OFF cells. As previously shown, CLU expression inversely correlates with that of MYCN [17]. Interestingly, we found that TCP treatment or LSD1 silencing de-represses CLU expression even in the presence of high MYCN (Figure 4A and 4B). To determine whether LSD1 is directly involved in binding and control of CLU expression, we performed ChIP assays under MYCN-ON and MYCN-OFF conditions. Results of Figure 4C and 4D demonstrate that both MYCN and LSD1 are recruited at the TSS and 1Kb chromatin regulatory regions of the CLU gene. As already observed for CDKN1A/p21, MYCN binding to CLU reflects its relative abundance (Figure 4C), whereas LSD1 inhibition or protein ablation by shLSD1 does not reduce MYCN binding to the CLU promoter sequences (Figure 4C). Alongside, LSD1 binds the CLU chromatin promoter as a function of MYCN abundance. Importantly, while LSD1 silencing evidently determines reduction of the protein occupancy at the gene promoter, TCP treatment does not have any effect on that (Figure 4D). Next, histone modifications occurring at the CLU promoter were also monitored by following the same criteria used for the CDKN1A/p21 gene. In the MYC-ON cells depletion of LSD1 enhances H3-acetylation (Figure 4E), whereas it lessens H3K27me3 (Figure 4F), consistent with the induction of CLU expression in these cells. High MYCN levels determine decreased levels of the H3K4Me2 (Figure 4G) that are not significantly affected by LSD1 inhibition. Overall our findings suggest that both MYCN and LSD1 bind to CLU promoter chromatin, and demonstrate that CLU expression is repressed by MYCN/LSD1 and that LSD1 inhibition is sufficient to relieve MYCN-driven Clusterin repression.

### Synergistic inhibition of NB cell growth by LSD1 and MYCN inhibitors

**Figure 5:**
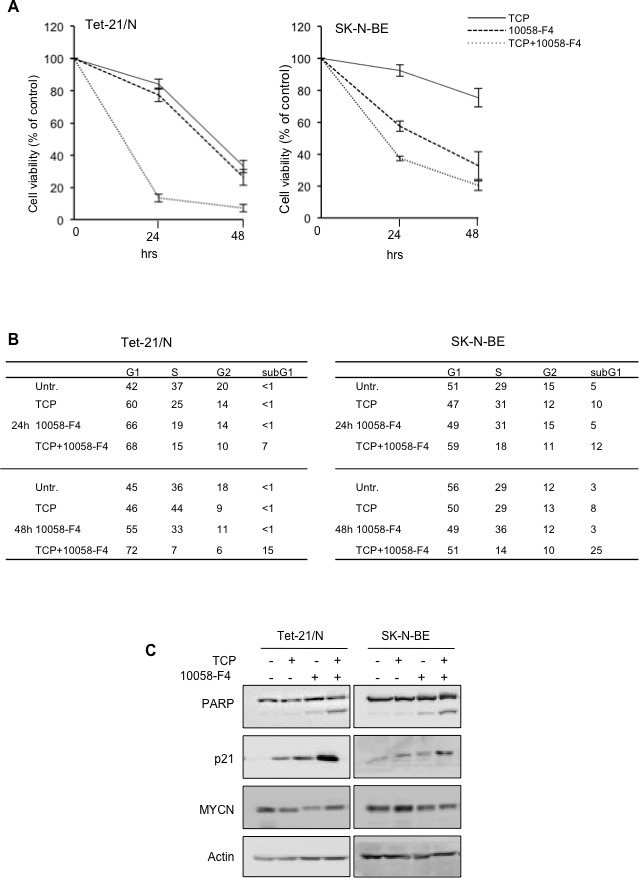
**A.** MTT assays of Tet-21/N and SK-N-BE cells treated with 1mM TCP, 75 μM 10058-F4, alone and in combination for 24 and 48 hours. Data from two independent experiments were used. **B.** Percentage of cell-cycle distribution of Tet-21/N and SK-N-BE (2) cells, treated with MYCN and LSD1 inhibitors as indicated, was measured by Flow cytometry analysis. Cells were treated with TCP and 10058-F4 for 24 and 48 hours and stained with Propidium Iodide for cell cycle profile; the average values from three independent experiments are reported in the tables; all standard deviations are <15%. **C.** LSD1 and MYCN inhibitors co-treatment increases apoptosis in NB cells. Western blotting of protein extract from Tet-21/N and SK-N-BE cells, treated with TCP, 10058-F4 or both for 48 hrs, using PARP (detecting both full length protein and cleaved fragment) p21 and MYCN antibodies. Actin has been probed as loading control.

The findings reported above strongly suggest that both MYCN and LSD1 cooperate to repress Neuroblastoma suppressor genes such as CDKN1A and CLU. Based on that, we wished to assess whether pharmacological inhibition of either MYCN or LSD1 or combination of both drugs, may have therapeutic relevance particularly in the context of MYCN-amplified cells. It has been recently reported that 10058-F4, a small molecule inhibitor of c-Myc, is also effective on MYCN protein by preventing MYCN/MAX dimerization [38]. We evaluated the effect of TCP and 10058-F4 compounds on cell growth of MYCN-ON Tet21/N and SK-N-BE (2), and we found that both molecules inhibited cell growth in time and concentration dependent manner (Supplementary Figure 1). To determine the effects of both drugs on NB cells viability, cells were treated with the highest concentration of TCP and 10058-F4 that resulted effective in viability reduction without resulting in excessive cell death (Supplementary Fig. 1). Data presented in Figure 5A show that both drugs affected cell viability and most notably, co-treatment with the two drugs cooperatively reduced viability in both cell lines. Next we assessed if treatment with single or combined drugs affects cell cycle distribution and apoptosis. Flow cytometry results shown in Figure 5B demonstrate that co-treatment with both drugs effectively causes a strong increase of the sub-G1 population suggesting the induction of apoptosis. Activation of apoptosis was then confirmed by analyzing PARP cleavage by Western blotting (Figure 5C). PARP cleavage was not observed in TCP treated cells, and barely detectable after 10058-F4 treatments. However, a robust increase of the cleaved PARP was observed in cells treated with combined drugs. Collectively these findings suggest that concomitant inhibition of both MYCN and LSD1 reduces neuroblastoma cell viability through activation of the apoptotic process.

## DISCUSSION

MYCN has a causative role in Neuroblastoma and its amplification correlates with poor prognosis. Several studies demonstrated that MYCN activates proliferation, cell cycle progression and promotes a stem like state blocking differentiation pathways [39]. The current model of MYCN functions in NB implies both transcription activation and repression of dedicated targets through interactions with transcription factors and histone modifiers [10]. Histone demethylase LSD1/KDM1a plays an important role in stem cell biology and tumorigenesis, especially in the maintenance of the silencing of differentiation genes [29, 32]. Accordingly, LSD1/KDM1a is involved in maintaining the undifferentiated, malignant phenotype of neuroblastoma cells. Inhibition of LSD1 induces differentiation of tumor cells into post-mitotic neurons and blocks neuroblastoma xenograft growth [18]. However, the molecular mechanisms underlying these properties are largely unknown. Here we report that LSD1 physically interacts with MYCN and that the LSD1/MYCN protein complex occupies MYCN target gene promoters. We show that LSD1 interacts with MYCN and functionally cooperates with MYCN in the repression of CDKN1A/p21 and CLU MYCN targets. CDKN1A/p21 is one of the major proteins involved in negative regulation of progression through the cell cycle while CLU is a multifunctional protein proposed to function as a tumor suppressor in Neuroblastoma. We demonstrate that both MYCN and LSD1 binds to chromatin promoter regions of CDKN1A/p21 and CLU, and that MYCN binding to these genes is not dependent upon LSD1 recruitment. Conversely, LSD1 binding was drastically reduced in cells expressing low levels of MYCN, suggesting that LSD1 recruitment might be dependent upon MYCN presence and/or abundance. Notably, we find that LSD1 inhibition is sufficient to restore CDKN1A/p21 and CLU expression in presence of high levels of MYCN, suggesting an important role of LSD1 in transcriptional repression of these MYCN targets. Collectively, these findings demonstrated that MYCN and LSD1 cooperate to repress CLU and p21 gene transcription.

Accordingly with the cooperative effects exerted by MYCN and LSD1 in keeping the repressive state of these two growth suppressor genes, we found that combined pharmacological inhibition of MYCN and LSD1 through the use of small molecule inhibitors of MYCN and LSD1 (TCP and 10058-F4) synergistically reduces Neuroblastoma cell viability *in vitro* through activation of the apoptotic process. This result is two-fold significative. On one hand the combination of LSD1 and MYCN inhibitors may have strong therapeutic relevance in the context of MYCN-driven Neuroblastoma. On the other hand it provides hints on the mechanism by which the MYCN/LSD1 complex can exert its transcriptional effect. It may appear that MYCN just serves as a recruiting platform for LSD1. In this case however the displacement of the platform by the MYCN inhibitor would be sufficient to render LSD1 inoperative with or without TCP. The fact that TCP synergistically cooperates with the MYCN inhibitor suggests that the MYCN and LSD1 engagement is of a particular nature; indeed, MYCN may exert novel functions beyond the simple recruitment. This phenomenon may be related to the fact that MYCN and LSD1 operate in the context of different repressive complexes. For example we have previously showed that MYCN represses CDKN1A/p21 also through the transcription factors MIZ-1 and SP1 [15] whereas it represses CLU expression by recruiting the Polycomb member EZH2 [17]. Furthermore, alternative splicing of LSD1 have been shown to modulate neurite morphogenesis [40]. While this manuscript was in preparation Laurent et al showed that a specific LSD1 isoform can regulate neuronal differentiation [41]; the role of different isoforms in MYCN/LSD1 complexes remains to be explored. Taken together these findings point to the existence of multiple and distinct MYCN/LSD1 complexes which actuate a transcription repression program through definite mechanisms. Clearly further studies on a wider set of MYCN/LSD1 common target genes will be instrumental to address these issues and to determine the exact molecular background in which the LSD1/MYCN complex operates inside cancer cells.

In this study we explored the functional interaction between LSD1 and MYCN and how such an interaction may be critical for Neuroblastoma biology. Results highlight a complex scenario in which the cooperation between LSD1 and MYCN is exerted at different and distinct levels and strongly impacts on MYCN-driven oncogenesis. The possibility to specifically inhibit the function of both proteins is of great importance since it provides the bases to the design and development of novel therapeutic approaches to treat MYCN-induced cancers.

## MATERIALS AND METHODS

### Cell culture and drug treatments

Human HEK 293T and SHEP Tet-21/N cells were cultured in Dulbecco's modified Eagle's Medium (DMEM) supplemented with antibiotics, 10% fetal calf serum. SK-N-BE (2) was cultured in 1:1 mixture DMEM/F-12 containing 10% FBS. All cell lines were incubated at 37°C in humidified atmosphere with 5% CO_2_. When indicated, cells were treated with TCP (0,3mM/ 0,6mM/ 1mM, Enzo Life Sciences), 10058-F4 (35μM /50μM /75μM, Sigma) or both (1mM+ 75μM) for 12, 24 or 48 hrs. For 3-(4,5-Dimethylthiazol-2-yl)-2,5-diphenyltetrazolium bromide cell proliferation (MTT) assay cells were seeded at a density of 2,500 per well and cultured in standard medium, replaced daily. MTT assay was performed according to the manufacture's protocol (Roche).

### Flow cytometry analysis

Cell treated as described were pelleted by centrifugation and resuspended at 1 × 10^6 cells/mL in Ethanol 70% in PBS at 4°C for one overnight for fixation. Then, 2 × 10^6 cells were permeabilized with 0,1% Triton X-100/PBS for 15′, blocked in 5% Bovine Serum Albumin/PBS and and stained with 2,5 μg/mL Propidium Iodide for 1hr. Cells were characterized by using a FACS Calibur (BD) and the data analyzed by Cell Quest Software and Cyflogic softwares.

### Western blot and co-immunoprecipitation

1,5 × 10^6^ cells treated in different experiments were lysed with buffer F (10 m1 Tris-HCl pH 7.5, 150 mM NaCl, 30 mM Na_4_O_7_P_2_, 50 mM NaF, 5 mM ZnCl_2_, 0.1 mM Na_3_VO_4_, 1% Triton, 0.1mM PMSF). 50 μg of protein extracts were loaded and separated by SDS-PAGE and Western blot was performed with following antibodies: MYCN (sc-53993, Santa Cruz), LSD1 (ab17721, Abcam), p21 (sc-397, Santa Cruz), p53 (sc-126, Santa Cruz), Clusterin-α (sc-6420, Santa Cruz), PARP-1 (sc-53643, Santa Cruz), Actin (sc-1616, Santa Cruz), α-actinin (sc-17829, Santa Cruz). Co-immunoprecipitation were performed using Tet-21/N and in HEK 293T using MYCN antibody (sc-53993, Santa Cruz). Tet-21/N cells are cultivated with (MYCN OFF) or without (MYC ON) tetracyclin (6 days). HEK 293T cells were transiently co-transfected with 3xFLAG-LSD1 and different MYCN mutants [42] by the polyethylenimine (PEI 25 K) method. Protein extracts from 1 mg of Tet-21/N cells or 0,3mg of HEK 293T transfected cells were incubated with MYCN antibody and processed as previously described [42]. All interactions were carried out overnight at 4°C. After incubation, the beads were washed at least five times using buffer F before loading on SDS–PAGE. Protein interactions were assessed by immunoblotting using the following antibodies: MYCN (sc-53993, Santa Cruz), LSD1 (ab17721, Abcam), MAX (sc-197, Santa Cruz).

### GST Pull-down assay

The different GST-MYCN deletion mutants were previously reported [17]. Recombinant proteins were expressed in BL21 Escherichia coli cells, purified, and immobilized onto glutathione-agarose beads (Sigma-Aldrich). GST beads were then incubated with equal amounts of extract prepared from 293T transfected with the recombinant vector 3xFLAG-LSD1 [42]. Purified complexes were separated on SDS-PAGE and analyzed by Western blotting using anti-LSD1 antibody (F1804 - Sigma-Aldrich) and anti GST (G7781 - Sigma-Aldrich).

### qRT-PCR

RNA was extracted from Tet-21/N cells using EuroGold Trifast (EuroClone). cDNA was generated using Quantitec Reverse Transcription Kit (Qiagen), according to manufacturer's protocol. Quantitative analysis was performed using SYBR Green 2X PCR Master Mix (Applied Biosystem). Each sample was run in triplicate and normalized to the expression of housekeeping beta-glucoronidase (GUS) gene as previously described (34). Primers presented in Supplementary Table 1

### Chromatin immunoprecipitation

Chromatin assays were performed as described [18, 43]. Briefly 1×10^7^ cells were cross-linked using formaldehyde to a final concentration of 1% and reaction was stopped using 0.125M Glycine. Cell pellet was resuspended in Cell Lysis Buffer and after 6000rpm centrifugation RIPA buffer were added to perform nuclei lysis. DNA shearing was conducted by sonication using Bioruptor (Diagenode). A small aliquot of sonicated material was put aside and remaining sample immunoprecipitated using 5 micrograms of ChIP-grade antibodies. Rec-sepharose Protein A or G beads (Invitrogen) were used to immobilize immuno-complexes and after RNAse-A treatment (37°C 1 hour) reverse cross-linking were performed using Proteinase K (Roche) for 6 hours at 65°C. Immunoprecipitated DNA was purified using Phenol/Chloroform and Ethanol precipitation techniques. Antibodies used in this study were as follows: MYCN (B8.4.B, Santa Cruz Biotechnology), LSD1 (ab17721, ABCAM), H3K4me2 (07-030, Millipore), H3K27me3 (07-449, Millipore), H3Ac (06-599, Millipore). DNA was analyzed by qPCR using Primers presented in Supplementary Table 1

### siRNA treatments, Sh-RNA production and silencing assays

20 or 100nM siRNA targeting LSD1 (GE Dharmacon) or scramble were transfected in Tet-21/N cells using a MicroPorator Digital Bio Technology, in according to the protocol described in ref [34]. In Tet-21/N cells MYCN was turned off by the addiction of tetracycline (1μg/ml) for one week before treatment. In ChIP analysis sh-RNA silencing was performed as described [43]. Briefly, virus production was carried out on HEK 293T cells transfected (Effectene QIAGEN) with packaging vectors, pMD2.G (#12259 - Addgene) and psPAX2 (#12260 - Addgene), and pLKO.1 TRC ShRNA backbone plasmids. pLKO.1 TRC Lentiviral Non-targeting ShRNA control (#RHS6848) and pLKO.1 TRC Lentiviral ShRNA LSD1 (Clone ID-TRCN0000046068) were purchased at Open Biosystems-GE Dharmacon. Optimization experiment (1–100 multiplicity of infection, MOI) was carried out on Tet-21/N cells using puromycin kill curve (1μg/ml) and set at MOI 10. For shRNA Chip experiments Tet-21/N cells were transduced for 6 hours with MOI 10 and polybrene concentration set at 10μg/ml, selected with puromycin for 24 hours and then incubated for 24 hours with complete media without puromycin selection.

## SUPPLEMENTARY MATERIAL TABLE AND FIGURE


