# Supplementary material for: Lysine-specific demethylase (LSD1/KDM1A) and MYCN cooperatively repress tumor suppressor genes in neuroblastoma
Source: Oncotarget. 2015 May 4;6(16):14572–83. doi: 10.18632/oncotarget.3990 (PMC4546488; doi:10.18632/oncotarget.3990)
Supplement: Supplementary file 1 [file oncotarget-06-14572-s001.pdf]

Lysine-specific demethylase (LSD1/KDM1A) and MYCN cooperatively repress tumor suppressor genes in neuroblastoma

Supplementary Material

Supplementary Table 1 : **Primers used in qRT-PCR and qChIP**

	Gene (Distance from TSS)	primer FW	primer REV
qChIP	CDKN1A (-2,2 Kb)	GCTGGTGGCTATTTTGTCCTTG	TGGCAGATCACATACCCTGTTC
qChIP	CDKN1A (0 Kb)	TGGCAGATCACATACCCTGTTC	CTCTCTCACCTCCTCTGAGTGCC
qChIP	CDKN1A (-3.3Kb)	CCAGCTGGCTGATGTTAACAAC	TGGTCATCACACCTGCTATGTC
qChIP	CLU (-1 Kb)	TCCATAGTCCTGATCCTGAACTG	TTTGGAGCCAGGGATGTTTAAG
qChIP	CLU (0 Kb)	TTGAGCAGAGCCACACCAGGAC	TGCGAGCTGTGTCATCCCTCTC
qChIP	CLU (+1 Kb)	GTGGAGCATTGGGCACAACTG	CCAGAGGCAAAGGTTAGCACTG
qRT-PCR	MYCN	CACAAGGCCCTCAGTACCT	TGACCACGTCGATTTCTTCCT
qRT-PCR	CDKN1A	TCACTGTCTTGTACCCTTGTGC	GGCGTTTGGAGTGGTAGAAA
qRT-PCR	CLU	GAGCAGAGCGCTATAAATACGG	CCAATTCTGGAGTCTTTGCAC
qRT-PCR	GUSB	GTGGGCATTGTGCTACCTC	ATTTTTGTCCCGGCGAAC

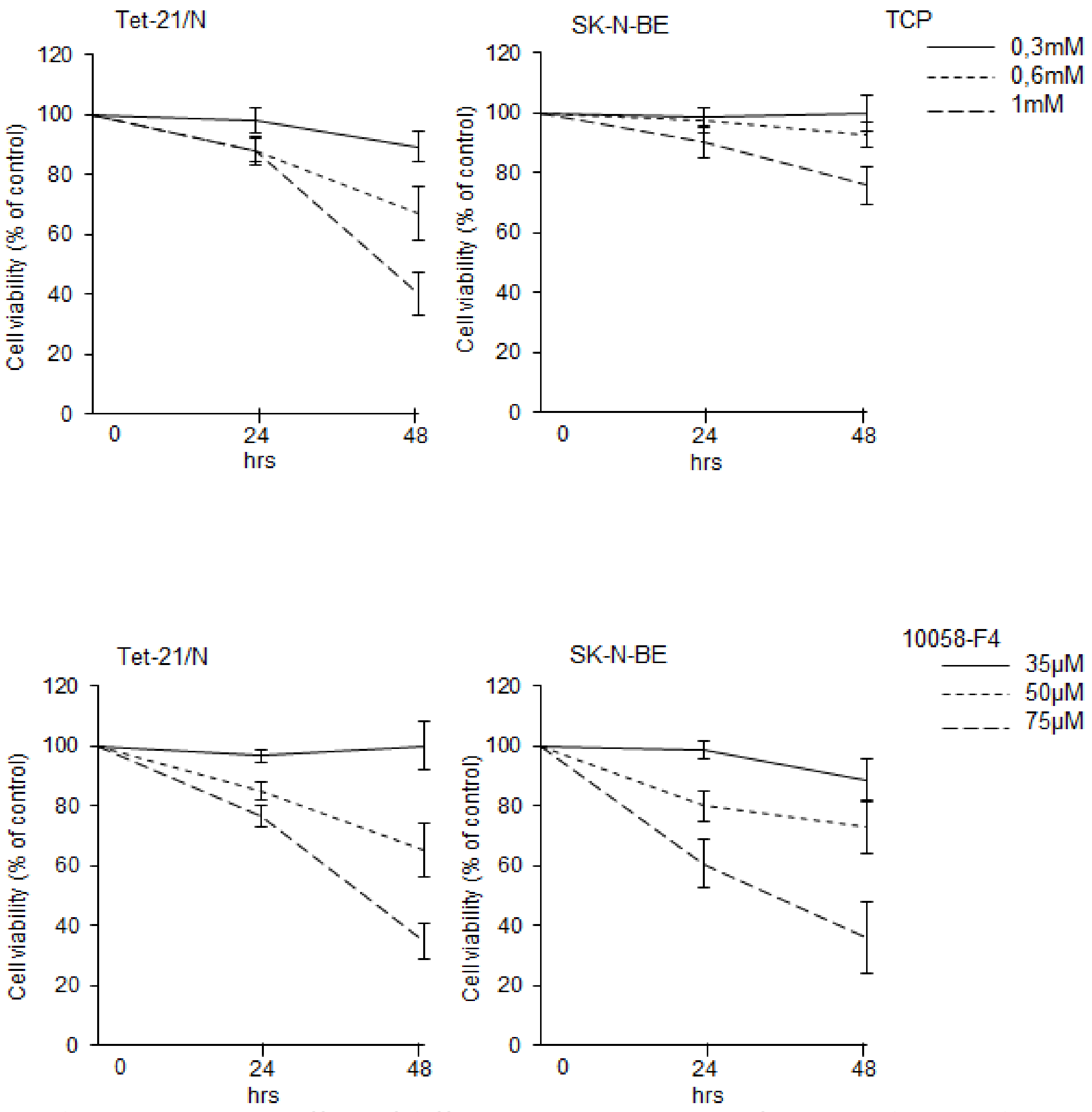

Supplementary Fig.1: Effect of different concentrations of LSD1 and MYC inhibitors on cell viability. MTT assays of Tet-21/N and SK-N-BE cells treated with 0,3, 0,6 and 1mM TCP, and 35, 50 and 75 μM 10058-F4 for 24 and 48 hours, as indicated. The result is an average of three independent experiments. Error bars indicate standard deviation.
